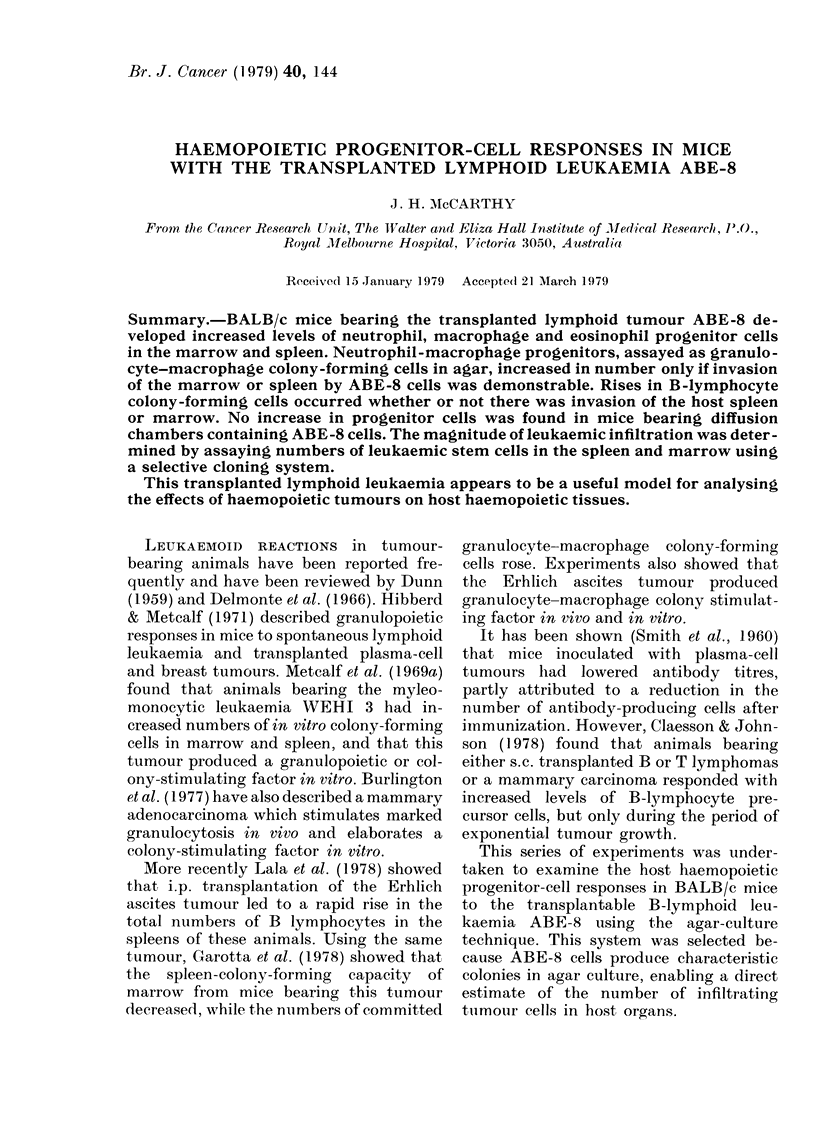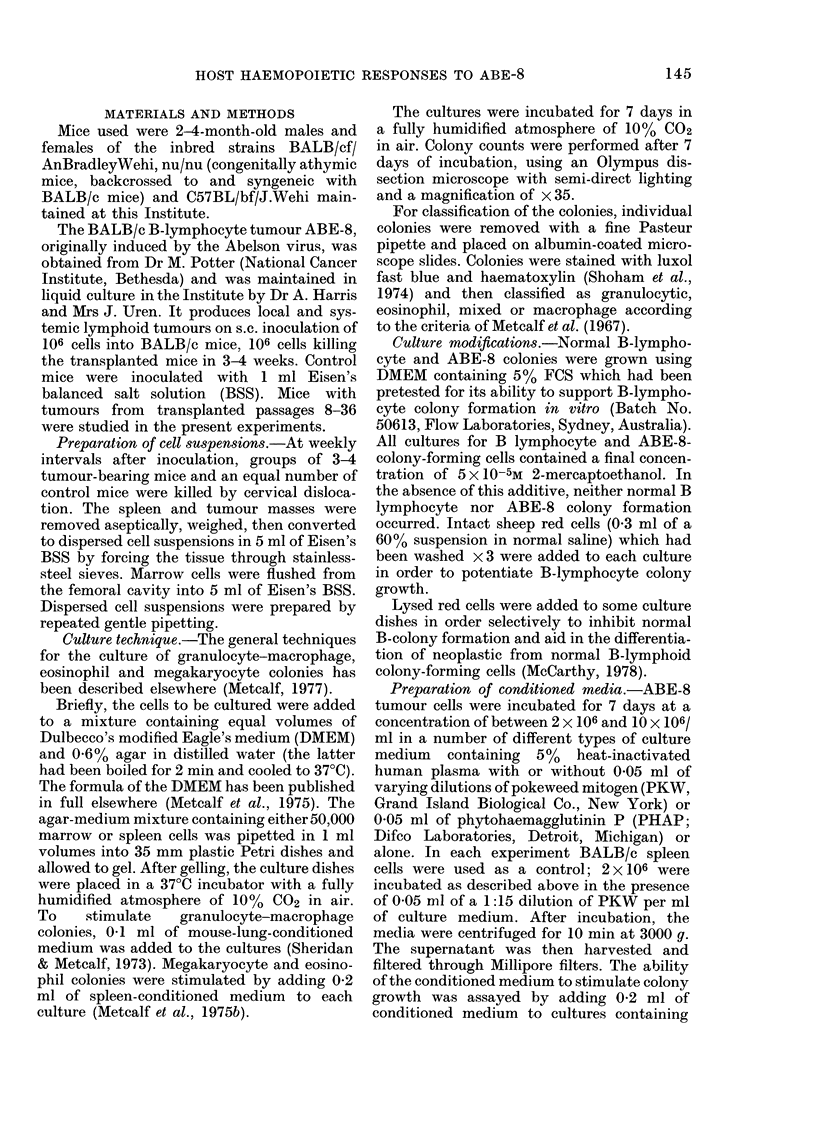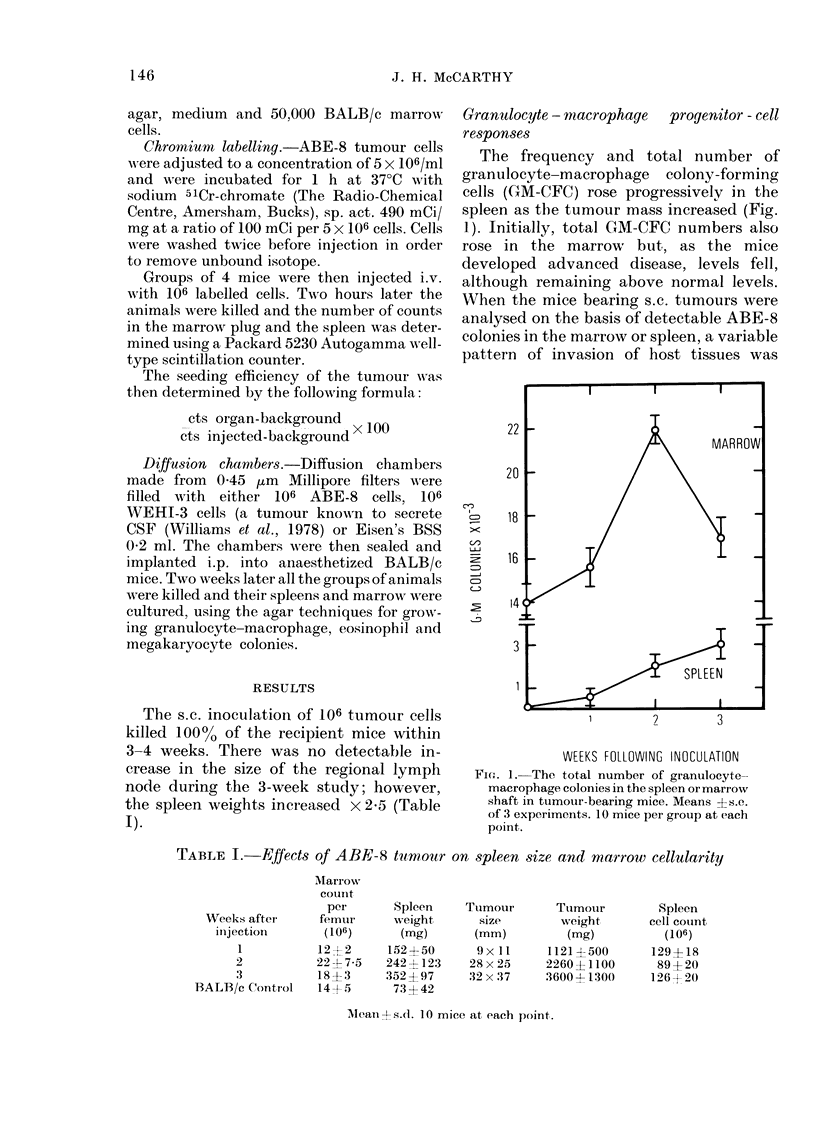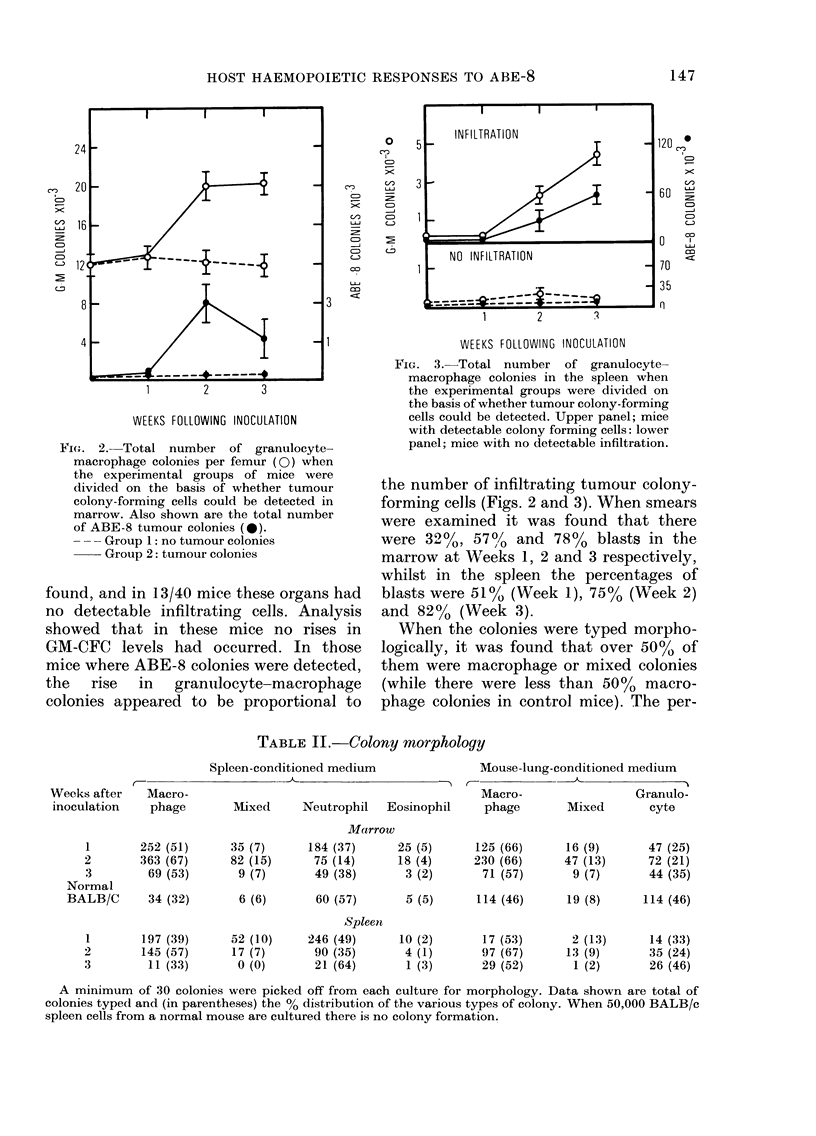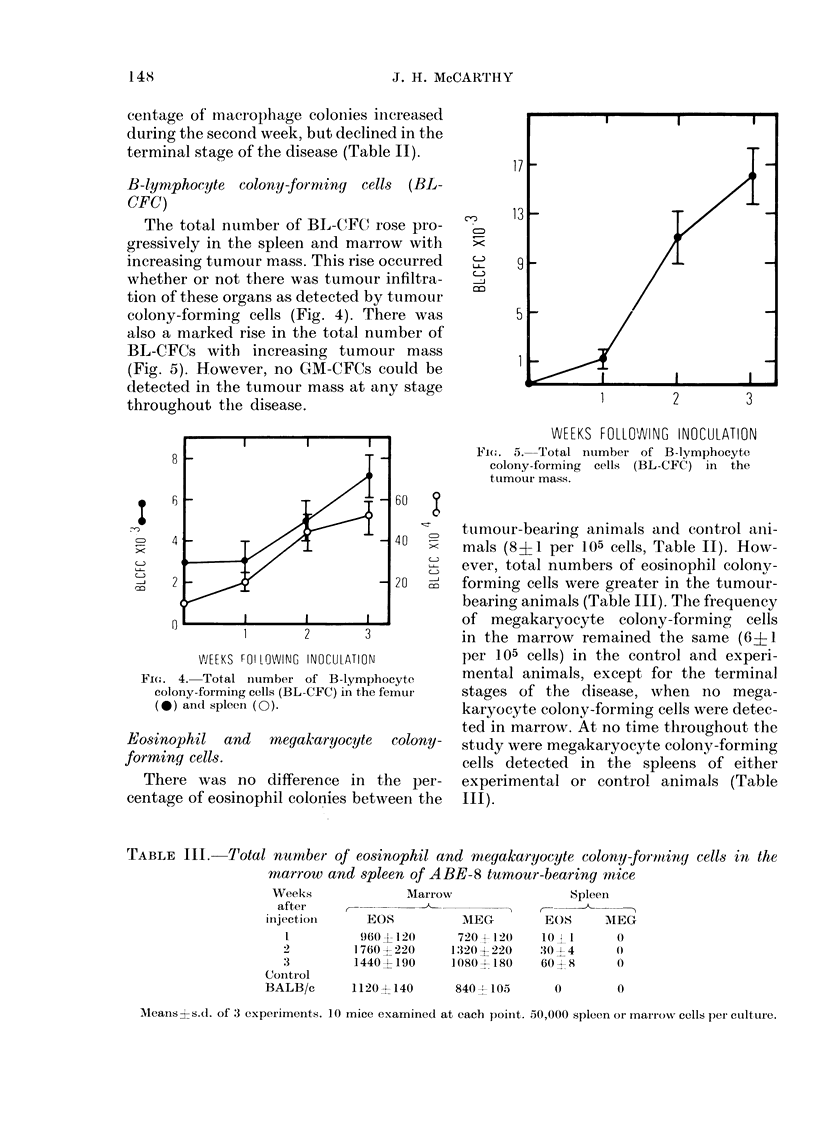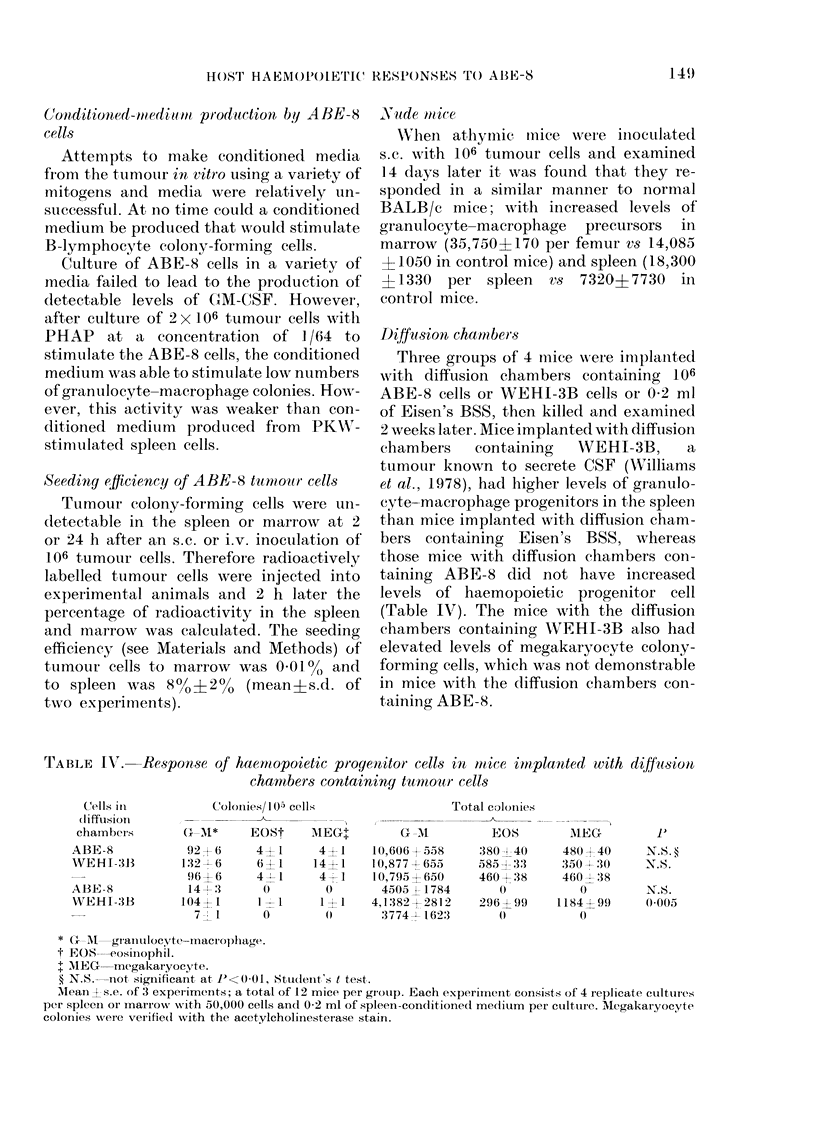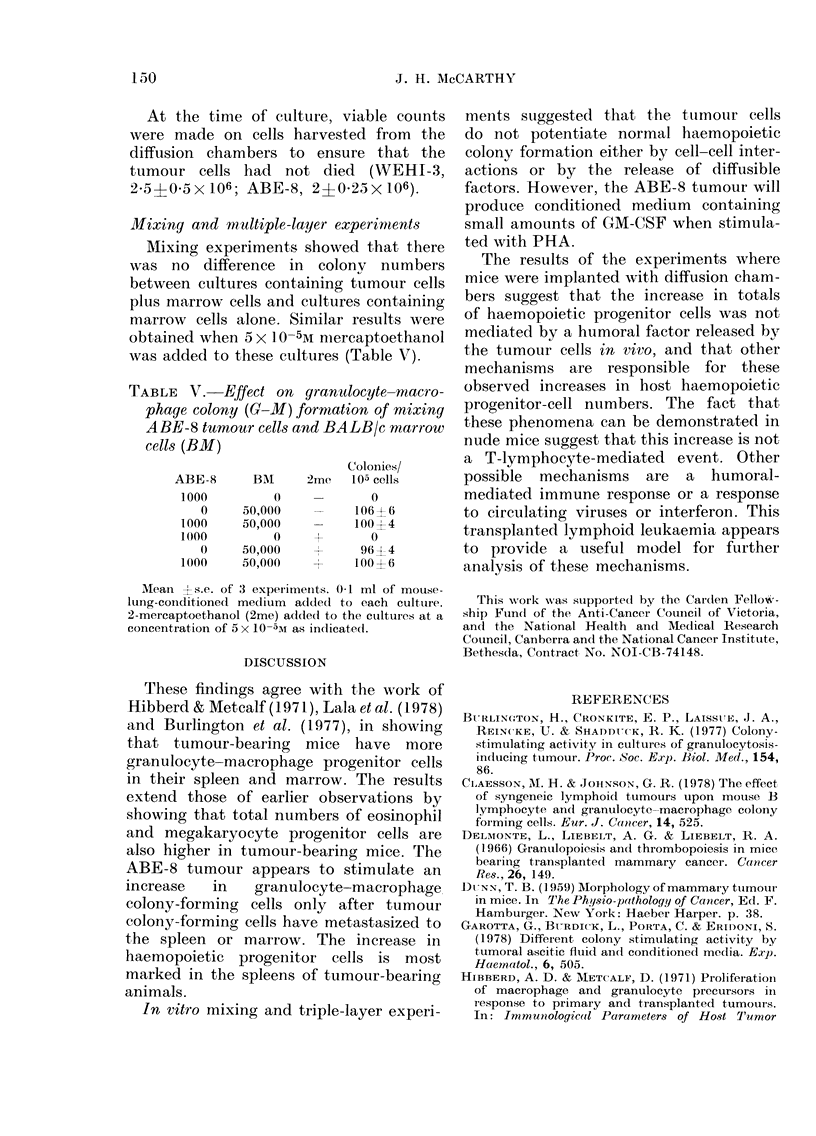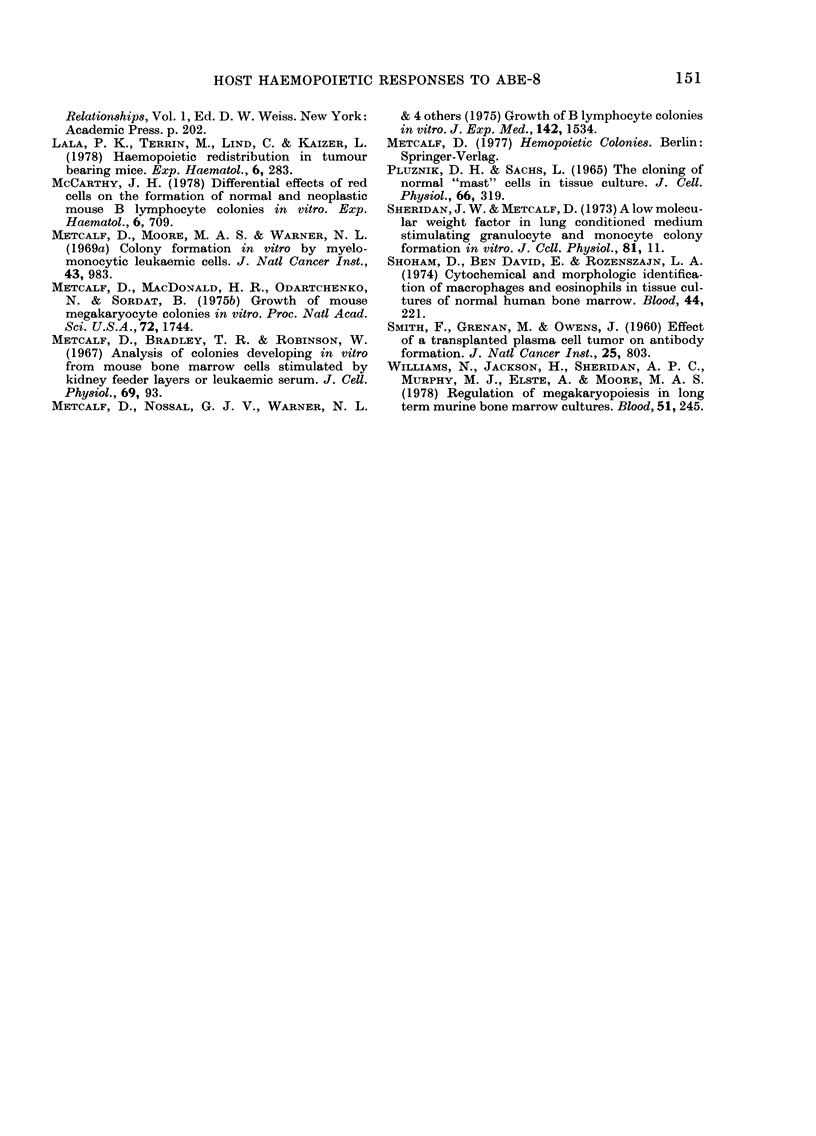# Haemopoietic progenitor-cell responses in mice with the transplanted lymphoid leukaemia ABE-8.

**DOI:** 10.1038/bjc.1979.150

**Published:** 1979-07

**Authors:** J. H. McCarthy

## Abstract

BALB/c mice bearing the transplanted lymphoid tumour ABE-8 developed increased levels of neutrophil, macrophage and eosinophil progenitor cells in the marrow and spleen. Neutrophil-macrophage progenitors, assayed as granulocyte-macrophage colony-forming cells in agar, increased in number only if invasion of the marrow or spleen by ABE-8 cells was demonstrable. Rises in B-lymphocyte colony-forming cells occurred whether or not there was invasion of the host spleen or marrow. No increase in progenitor cells was found in mice bearing diffusion chambers containing ABE-8 cells. The magnitude of leukaemic infiltration was determined by assaying numbers of leukaemic stem cells in the spleen and marrow using a selective cloning system. This transplanted lymphoid leukaemia appears to be a useful model for analysing the effects of haemopoietic tumours on host haemopoietic tissues.


*Br. J. Cancer* (1979) **40,** 144

### HAEMOPOIETIC PROGENITOR-CELL RESPONSES IN MICE WITH THE TRANSPLANTED LYMPHOID LEUKAEMIA ABE-8

J. H. McCARTHY

*From the Cancer Research Unit, The Walter and Eliza Hall Institute of Medical Research, P.O., Royal Melbourne Hospital, Victoria 3050, Australia*

Received 15 January 1979 Accepted 21 March 1979

**Summary.—BALB/c mice bearing the transplanted lymphoid tumour ABE-8 developed increased levels of neutrophil, macrophage and eosinophil progenitor cells in the marrow and spleen. Neutrophil-macrophage progenitors, assayed as granulocyte–macrophage colony-forming cells in agar, increased in number only if invasion of the marrow or spleen by ABE-8 cells was demonstrable. Rises in B-lymphocyte colony-forming cells occurred whether or not there was invasion of the host spleen or marrow. No increase in progenitor cells was found in mice bearing diffusion chambers containing ABE-8 cells. The magnitude of leukaemic infiltration was determined by assaying numbers of leukaemic stem cells in the spleen and marrow using a selective cloning system.**

**This transplanted lymphoid leukaemia appears to be a useful model for analysing the effects of haemopoietic tumours on host haemopoietic tissues.**

LEUKAEMOID REACTIONS in tumour-bearing animals have been reported frequently and have been reviewed by Dunn (1959) and Delmonte *et al.* (1966). Hibberd & Metcalf (1971) described granulopoietic responses in mice to spontaneous lymphoid leukaemia and transplanted plasma-cell and breast tumours. Metcalf *et al.* (1969*a*) found that animals bearing the myleomonocytic leukaemia WEHI 3 had increased numbers of *in vitro* colony-forming cells in marrow and spleen, and that this tumour produced a granulopoietic or colony-stimulating factor *in vitro*. Burlington *et al.* (1977) have also described a mammary adenocarcinoma which stimulates marked granulocytosis *in vivo* and elaborates a colony-stimulating factor *in vitro*.

More recently Lala *et al.* (1978) showed that i.p. transplantation of the Erhlich ascites tumour led to a rapid rise in the total numbers of B lymphocytes in the spleens of these animals. Using the same tumour, Garotta *et al.* (1978) showed that the spleen-colony-forming capacity of marrow from mice bearing this tumour decreased, while the numbers of committed granulocyte–macrophage colony-forming cells rose. Experiments also showed that the Erhlich ascites tumour produced granulocyte–macrophage colony stimulating factor *in vivo* and *in vitro*.

It has been shown (Smith *et al.*, 1960) that mice inoculated with plasma-cell tumours had lowered antibody titres, partly attributed to a reduction in the number of antibody-producing cells after immunization. However, Claesson & Johnson (1978) found that animals bearing either s.c. transplanted B or T lymphomas or a mammary carcinoma responded with increased levels of B-lymphocyte precursor cells, but only during the period of exponential tumour growth.

This series of experiments was undertaken to examine the host haemopoietic progenitor-cell responses in BALB/c mice to the transplantable B-lymphoid leukaemia ABE-8 using the agar-culture technique. This system was selected because ABE-8 cells produce characteristic colonies in agar culture, enabling a direct estimate of the number of infiltrating tumour cells in host organs.

### MATERIALS AND METHODS

Mice used were 2–4-month-old males and females of the inbred strains BALB/cf/AnBradleyWehi, nu/nu (congenitally athymic mice, backcrossed to and syngeneic with BALB/c mice) and C57BL/bf/J.Wehi maintained at this Institute.

The BALB/c B-lymphocyte tumour ABE-8, originally induced by the Abelson virus, was obtained from Dr M. Potter (National Cancer Institute, Bethesda) and was maintained in liquid culture in the Institute by Dr A. Harris and Mrs J. Uren. It produces local and systemic lymphoid tumours on s.c. inoculation of $10^6$ cells into BALB/c mice, $10^6$ cells killing the transplanted mice in 3–4 weeks. Control mice were inoculated with 1 ml Eisen's balanced salt solution (BSS). Mice with tumours from transplanted passages 8–36 were studied in the present experiments.

*Preparation of cell suspensions.*—At weekly intervals after inoculation, groups of 3–4 tumour-bearing mice and an equal number of control mice were killed by cervical dislocation. The spleen and tumour masses were removed aseptically, weighed, then converted to dispersed cell suspensions in 5 ml of Eisen's BSS by forcing the tissue through stainless-steel sieves. Marrow cells were flushed from the femoral cavity into 5 ml of Eisen's BSS. Dispersed cell suspensions were prepared by repeated gentle pipetting.

*Culture technique.*—The general techniques for the culture of granulocyte–macrophage, eosinophil and megakaryocyte colonies has been described elsewhere (Metcalf, 1977).

Briefly, the cells to be cultured were added to a mixture containing equal volumes of Dulbecco's modified Eagle's medium (DMEM) and 0·6% agar in distilled water (the latter had been boiled for 2 min and cooled to 37°C). The formula of the DMEM has been published in full elsewhere (Metcalf *et al.*, 1975). The agar-medium mixture containing either 50,000 marrow or spleen cells was pipetted in 1 ml volumes into 35 mm plastic Petri dishes and allowed to gel. After gelling, the culture dishes were placed in a 37°C incubator with a fully humidified atmosphere of 10% $CO_2$ in air. To stimulate granulocyte–macrophage colonies, 0·1 ml of mouse-lung-conditioned medium was added to the cultures (Sheridan & Metcalf, 1973). Megakaryocyte and eosinophil colonies were stimulated by adding 0·2 ml of spleen-conditioned medium to each culture (Metcalf *et al.*, 1975*b*).

The cultures were incubated for 7 days in a fully humidified atmosphere of 10% $CO_2$ in air. Colony counts were performed after 7 days of incubation, using an Olympus dissection microscope with semi-direct lighting and a magnification of ×35.

For classification of the colonies, individual colonies were removed with a fine Pasteur pipette and placed on albumin-coated microscope slides. Colonies were stained with luxol fast blue and haematoxylin (Shoham *et al.*, 1974) and then classified as granulocytic, eosinophil, mixed or macrophage according to the criteria of Metcalf *et al.* (1967).

*Culture modifications.*—Normal B-lymphocyte and ABE-8 colonies were grown using DMEM containing 5% FCS which had been pretested for its ability to support B-lymphocyte colony formation *in vitro* (Batch No. 50613, Flow Laboratories, Sydney, Australia). All cultures for B lymphocyte and ABE-8-colony-forming cells contained a final concentration of $5 \times 10^{-5}$M 2-mercaptoethanol. In the absence of this additive, neither normal B lymphocyte nor ABE-8 colony formation occurred. Intact sheep red cells (0·3 ml of a 60% suspension in normal saline) which had been washed ×3 were added to each culture in order to potentiate B-lymphocyte colony growth.

Lysed red cells were added to some culture dishes in order selectively to inhibit normal B-colony formation and aid in the differentiation of neoplastic from normal B-lymphoid colony-forming cells (McCarthy, 1978).

*Preparation of conditioned media.*—ABE-8 tumour cells were incubated for 7 days at a concentration of between $2 \times 10^6$ and $10 \times 10^6$/ml in a number of different types of culture medium containing 5% heat-inactivated human plasma with or without 0·05 ml of varying dilutions of pokeweed mitogen (PKW, Grand Island Biological Co., New York) or 0·05 ml of phytohaemagglutinin P (PHAP; Difco Laboratories, Detroit, Michigan) or alone. In each experiment BALB/c spleen cells were used as a control; $2 \times 10^6$ were incubated as described above in the presence of 0·05 ml of a 1:15 dilution of PKW per ml of culture medium. After incubation, the media were centrifuged for 10 min at 3000 *g*. The supernatant was then harvested and filtered through Millipore filters. The ability of the conditioned medium to stimulate colony growth was assayed by adding 0·2 ml of conditioned medium to cultures containing agar, medium and 50,000 BALB/c marrow cells.

*Chromium labelling.*—ABE-8 tumour cells were adjusted to a concentration of $5 \times 10^6$/ml and were incubated for 1 h at 37°C with sodium $^{51}$Cr-chromate (The Radio-Chemical Centre, Amersham, Bucks), sp. act. 490 mCi/mg at a ratio of 100 mCi per $5 \times 10^6$ cells. Cells were washed twice before injection in order to remove unbound isotope.

Groups of 4 mice were then injected i.v. with $10^6$ labelled cells. Two hours later the animals were killed and the number of counts in the marrow plug and the spleen was determined using a Packard 5230 Autogamma well-type scintillation counter.

The seeding efficiency of the tumour was then determined by the following formula:

$$\frac{\text{cts organ-background}}{\text{cts injected-background}} \times 100$$

*Diffusion chambers.*—Diffusion chambers made from 0·45 $\mu$m Millipore filters were filled with either $10^6$ ABE-8 cells, $10^6$ WEHI-3 cells (a tumour known to secrete CSF (Williams *et al.*, 1978) or Eisen's BSS 0·2 ml. The chambers were then sealed and implanted i.p. into anaesthetized BALB/c mice. Two weeks later all the groups of animals were killed and their spleens and marrow were cultured, using the agar techniques for growing granulocyte–macrophage, eosinophil and megakaryocyte colonies.

### RESULTS

The s.c. inoculation of $10^6$ tumour cells killed 100% of the recipient mice within 3–4 weeks. There was no detectable increase in the size of the regional lymph node during the 3-week study; however, the spleen weights increased $\times 2·5$ (Table I).

*Granulocyte – macrophage progenitor - cell responses*

The frequency and total number of granulocyte–macrophage colony-forming cells (GM-CFC) rose progressively in the spleen as the tumour mass increased (Fig. 1). Initially, total GM-CFC numbers also rose in the marrow but, as the mice developed advanced disease, levels fell, although remaining above normal levels. When the mice bearing s.c. tumours were analysed on the basis of detectable ABE-8 colonies in the marrow or spleen, a variable pattern of invasion of host tissues was

FIG. 1.—The total number of granulocyte–macrophage colonies in the spleen or marrow shaft in tumour-bearing mice. Means ±s.e. of 3 experiments. 10 mice per group at each point.

TABLE I.—*Effects of ABE-8 tumour on spleen size and marrow cellularity*

| Weeks after injection | Marrow count per femur ($10^6$) | Spleen weight (mg) | Tumour size (mm) | Tumour weight (mg) | Spleen cell count ($10^6$) |
|---|---|---|---|---|---|
| 1 | 12±2 | 152±50 | 9×11 | 1121±500 | 129±18 |
| 2 | 22±7·5 | 242±123 | 28×25 | 2260±1100 | 89±20 |
| 3 | 18±3 | 352±97 | 32×37 | 3600±1300 | 126±20 |
| BALB/c Control | 14±5 | 73±42 | | | |

Mean±s.d. 10 mice at each point.

FIG. 2.—Total number of granulocyte–macrophage colonies per femur (○) when the experimental groups of mice were divided on the basis of whether tumour colony-forming cells could be detected in marrow. Also shown are the total number of ABE-8 tumour colonies (●).
– – – Group 1: no tumour colonies
—— Group 2: tumour colonies

FIG. 3.—Total number of granulocyte–macrophage colonies in the spleen when the experimental groups were divided on the basis of whether tumour colony-forming cells could be detected. Upper panel; mice with detectable colony forming cells: lower panel; mice with no detectable infiltration.

found, and in 13/40 mice these organs had no detectable infiltrating cells. Analysis showed that in these mice no rises in GM-CFC levels had occurred. In those mice where ABE-8 colonies were detected, the rise in granulocyte–macrophage colonies appeared to be proportional to the number of infiltrating tumour colony-forming cells (Figs. 2 and 3). When smears were examined it was found that there were 32%, 57% and 78% blasts in the marrow at Weeks 1, 2 and 3 respectively, whilst in the spleen the percentages of blasts were 51% (Week 1), 75% (Week 2) and 82% (Week 3).

When the colonies were typed morphologically, it was found that over 50% of them were macrophage or mixed colonies (while there were less than 50% macrophage colonies in control mice). The per-

TABLE II.—*Colony morphology*

| | Spleen-conditioned medium | | | | Mouse-lung-conditioned medium | | |
|---|---|---|---|---|---|---|---|
| Weeks after inoculation | Macrophage | Mixed | Neutrophil | Eosinophil | Macrophage | Mixed | Granulocyte |
| | | | *Marrow* | | | | |
| 1 | 252 (51) | 35 (7) | 184 (37) | 25 (5) | 125 (66) | 16 (9) | 47 (25) |
| 2 | 363 (67) | 82 (15) | 75 (14) | 18 (4) | 230 (66) | 47 (13) | 72 (21) |
| 3 | 69 (53) | 9 (7) | 49 (38) | 3 (2) | 71 (57) | 9 (7) | 44 (35) |
| Normal BALB/C | 34 (32) | 6 (6) | 60 (57) | 5 (5) | 114 (46) | 19 (8) | 114 (46) |
| | | | *Spleen* | | | | |
| 1 | 197 (39) | 52 (10) | 246 (49) | 10 (2) | 17 (53) | 2 (13) | 14 (33) |
| 2 | 145 (57) | 17 (7) | 90 (35) | 4 (1) | 97 (67) | 13 (9) | 35 (24) |
| 3 | 11 (33) | 0 (0) | 21 (64) | 1 (3) | 29 (52) | 1 (2) | 26 (46) |

A minimum of 30 colonies were picked off from each culture for morphology. Data shown are total of colonies typed and (in parentheses) the % distribution of the various types of colony. When 50,000 BALB/c spleen cells from a normal mouse are cultured there is no colony formation.

centage of macrophage colonies increased during the second week, but declined in the terminal stage of the disease (Table II).

*B-lymphocyte colony-forming cells (BL-CFC)*

The total number of BL-CFC rose progressively in the spleen and marrow with increasing tumour mass. This rise occurred whether or not there was tumour infiltration of these organs as detected by tumour colony-forming cells (Fig. 4). There was also a marked rise in the total number of BL-CFCs with increasing tumour mass (Fig. 5). However, no GM-CFCs could be detected in the tumour mass at any stage throughout the disease.

FIG. 4.—Total number of B-lymphocyte colony-forming cells (BL-CFC) in the femur (●) and spleen (○).

*Eosinophil and megakaryocyte colony-forming cells.*

There was no difference in the percentage of eosinophil colonies between the tumour-bearing animals and control animals ($8 \pm 1$ per $10^5$ cells, Table II). However, total numbers of eosinophil colony-forming cells were greater in the tumour-bearing animals (Table III). The frequency of megakaryocyte colony-forming cells in the marrow remained the same ($6 \pm 1$ per $10^5$ cells) in the control and experimental animals, except for the terminal stages of the disease, when no megakaryocyte colony-forming cells were detected in marrow. At no time throughout the study were megakaryocyte colony-forming cells detected in the spleens of either experimental or control animals (Table III).

FIG. 5.—Total number of B-lymphocyte colony-forming cells (BL-CFC) in the tumour mass.

TABLE III.—*Total number of eosinophil and megakaryocyte colony-forming cells in the marrow and spleen of ABE-8 tumour-bearing mice*

| Weeks after injection | Marrow | | Spleen | |
|---|---|---|---|---|
| | EOS | MEG | EOS | MEG |
| 1 | $960 \pm 120$ | $720 \pm 120$ | $10 \pm 1$ | 0 |
| 2 | $1760 \pm 220$ | $1320 \pm 220$ | $30 \pm 4$ | 0 |
| 3 | $1440 \pm 190$ | $1080 \pm 180$ | $60 \pm 8$ | 0 |
| Control BALB/c | $1120 \pm 140$ | $840 \pm 105$ | 0 | 0 |

Means ± s.d. of 3 experiments. 10 mice examined at each point. 50,000 spleen or marrow cells per culture.

*Conditioned-medium production by ABE-8 cells*

Attempts to make conditioned media from the tumour *in vitro* using a variety of mitogens and media were relatively unsuccessful. At no time could a conditioned medium be produced that would stimulate B-lymphocyte colony-forming cells.

Culture of ABE-8 cells in a variety of media failed to lead to the production of detectable levels of GM-CSF. However, after culture of $2 \times 10^6$ tumour cells with PHAP at a concentration of 1/64 to stimulate the ABE-8 cells, the conditioned medium was able to stimulate low numbers of granulocyte–macrophage colonies. However, this activity was weaker than conditioned medium produced from PKW-stimulated spleen cells.

*Seeding efficiency of ABE-8 tumour cells*

Tumour colony-forming cells were undetectable in the spleen or marrow at 2 or 24 h after an s.c. or i.v. inoculation of $10^6$ tumour cells. Therefore radioactively labelled tumour cells were injected into experimental animals and 2 h later the percentage of radioactivity in the spleen and marrow was calculated. The seeding efficiency (see Materials and Methods) of tumour cells to marrow was 0·01% and to spleen was 8%±2% (mean±s.d. of two experiments).

*Nude mice*

When athymic mice were inoculated s.c. with $10^6$ tumour cells and examined 14 days later it was found that they responded in a similar manner to normal BALB/c mice; with increased levels of granulocyte–macrophage precursors in marrow (35,750±170 per femur *vs* 14,085 ±1050 in control mice) and spleen (18,300 ±1330 per spleen *vs* 7320±7730 in control mice.

*Diffusion chambers*

Three groups of 4 mice were implanted with diffusion chambers containing $10^6$ ABE-8 cells or WEHI-3B cells or 0·2 ml of Eisen's BSS, then killed and examined 2 weeks later. Mice implanted with diffusion chambers containing WEHI-3B, a tumour known to secrete CSF (Williams *et al.*, 1978), had higher levels of granulocyte–macrophage progenitors in the spleen than mice implanted with diffusion chambers containing Eisen's BSS, whereas those mice with diffusion chambers containing ABE-8 did not have increased levels of haemopoietic progenitor cell (Table IV). The mice with the diffusion chambers containing WEHI-3B also had elevated levels of megakaryocyte colony-forming cells, which was not demonstrable in mice with the diffusion chambers containing ABE-8.

TABLE IV.—*Response of haemopoietic progenitor cells in mice implanted with diffusion chambers containing tumour cells*

| Cells in diffusion chambers | Colonies/$10^5$ cells | | | Total colonies | | | |
|---|---|---|---|---|---|---|---|
| | G–M* | EOS† | MEG‡ | G–M | EOS | MEG | *P* |
| ABE-8 | 92±6 | 4±1 | 4±1 | 10,606±558 | 380±40 | 480±40 | N.S.§ |
| WEHI-3B | 132±6 | 6±1 | 14±1 | 10,877±655 | 585±33 | 350±30 | N.S. |
| — | 96±6 | 4±1 | 4±1 | 10,795±650 | 460±38 | 460±38 | |
| ABE-8 | 14±3 | 0 | 0 | 4505±1784 | 0 | 0 | N.S. |
| WEHI-3B | 104±1 | 1±1 | 1±1 | 4,1382±2812 | 296±99 | 1184±99 | 0·005 |
| — | 7±1 | 0 | 0 | 3774±1623 | 0 | 0 | |

* G–M—granulocyte–macrophage.
† EOS—eosinophil.
‡ MEG—megakaryocyte.
§ N.S.—not significant at $P < 0{\cdot}01$, Student's $t$ test.
Mean±s.e. of 3 experiments; a total of 12 mice per group. Each experiment consists of 4 replicate cultures per spleen or marrow with 50,000 cells and 0·2 ml of spleen-conditioned medium per culture. Megakaryocyte colonies were verified with the acetylcholinesterase stain.

At the time of culture, viable counts were made on cells harvested from the diffusion chambers to ensure that the tumour cells had not died (WEHI-3, $2 \cdot 5 \pm 0 \cdot 5 \times 10^6$; ABE-8, $2 \pm 0 \cdot 25 \times 10^6$).

*Mixing and multiple-layer experiments*

Mixing experiments showed that there was no difference in colony numbers between cultures containing tumour cells plus marrow cells and cultures containing marrow cells alone. Similar results were obtained when $5 \times 10^{-5}$M mercaptoethanol was added to these cultures (Table V).

TABLE V.—*Effect on granulocyte–macrophage colony (G–M) formation of mixing ABE-8 tumour cells and BALB/c marrow cells (BM)*

| ABE-8 | BM | 2me | Colonies/ $10^5$ cells |
|---|---|---|---|
| 1000 | 0 | – | 0 |
| 0 | 50,000 | – | $106 \pm 6$ |
| 1000 | 50,000 | – | $100 \pm 4$ |
| 1000 | 0 | + | 0 |
| 0 | 50,000 | + | $96 \pm 4$ |
| 1000 | 50,000 | + | $100 \pm 6$ |

Mean ± s.e. of 3 experiments. 0·1 ml of mouse-lung-conditioned medium added to each culture. 2-mercaptoethanol (2me) added to the cultures at a concentration of $5 \times 10^{-5}$M as indicated.

### DISCUSSION

These findings agree with the work of Hibberd & Metcalf (1971), Lala *et al.* (1978) and Burlington *et al.* (1977), in showing that tumour-bearing mice have more granulocyte–macrophage progenitor cells in their spleen and marrow. The results extend those of earlier observations by showing that total numbers of eosinophil and megakaryocyte progenitor cells are also higher in tumour-bearing mice. The ABE-8 tumour appears to stimulate an increase in granulocyte–macrophage colony-forming cells only after tumour colony-forming cells have metastasized to the spleen or marrow. The increase in haemopoietic progenitor cells is most marked in the spleens of tumour-bearing animals.

*In vitro* mixing and triple-layer experiments suggested that the tumour cells do not potentiate normal haemopoietic colony formation either by cell–cell interactions or by the release of diffusible factors. However, the ABE-8 tumour will produce conditioned medium containing small amounts of GM-CSF when stimulated with PHA.

The results of the experiments where mice were implanted with diffusion chambers suggest that the increase in totals of haemopoietic progenitor cells was not mediated by a humoral factor released by the tumour cells *in vivo*, and that other mechanisms are responsible for these observed increases in host haemopoietic progenitor-cell numbers. The fact that these phenomena can be demonstrated in nude mice suggest that this increase is not a T-lymphocyte-mediated event. Other possible mechanisms are a humoral-mediated immune response or a response to circulating viruses or interferon. This transplanted lymphoid leukaemia appears to provide a useful model for further analysis of these mechanisms.

This work was supported by the Carden Fellowship Fund of the Anti-Cancer Council of Victoria, and the National Health and Medical Research Council, Canberra and the National Cancer Institute, Bethesda, Contract No. NOI-CB-74148.

### REFERENCES

BURLINGTON, H., CRONKITE, E. P., LAISSUE, J. A., REINCKE, U. & SHADDUCK, R. K. (1977) Colony-stimulating activity in cultures of granulocytosis-inducing tumour. *Proc. Soc. Exp. Biol. Med.*, **154**, 86.

CLAESSON, M. H. & JOHNSON, G. R. (1978) The effect of syngeneic lymphoid tumours upon mouse B lymphocyte and granulocyte–macrophage colony forming cells. *Eur. J. Cancer*, **14**, 525.

DELMONTE, L., LIEBELT, A. G. & LIEBELT, R. A. (1966) Granulopoiesis and thrombopoiesis in mice bearing transplanted mammary cancer. *Cancer Res.*, **26**, 149.

DUNN, T. B. (1959) Morphology of mammary tumour in mice. In *The Physio-pathology of Cancer*, Ed. F. Hamburger. New York: Haeber Harper. p. 38.

GAROTTA, G., BURDICK, L., PORTA, C. & ERIDONI, S. (1978) Different colony stimulating activity by tumoral ascitic fluid and conditioned media. *Exp. Haematol.*, **6**, 505.

HIBBERD, A. D. & METCALF, D. (1971) Proliferation of macrophage and granulocyte precursors in response to primary and transplanted tumours. In: *Immunological Parameters of Host Tumor*

*Relationships*, Vol. 1, Ed. D. W. Weiss. New York: Academic Press. p. 202.

LALA, P. K., TERRIN, M., LIND, C. & KAIZER, L. (1978) Haemopoietic redistribution in tumour bearing mice. *Exp. Haematol.*, **6**, 283.

McCARTHY, J. H. (1978) Differential effects of red cells on the formation of normal and neoplastic mouse B lymphocyte colonies *in vitro*. *Exp. Haematol.*, **6**, 709.

METCALF, D., MOORE, M. A. S. & WARNER, N. L. (1969*a*) Colony formation *in vitro* by myelomonocytic leukaemic cells. *J. Natl Cancer Inst.*, **43**, 983.

METCALF, D., MACDONALD, H. R., ODARTCHENKO, N. & SORDAT, B. (1975*b*) Growth of mouse megakaryocyte colonies *in vitro*. *Proc. Natl Acad. Sci. U.S.A.*, **72**, 1744.

METCALF, D., BRADLEY, T. R. & ROBINSON, W. (1967) Analysis of colonies developing *in vitro* from mouse bone marrow cells stimulated by kidney feeder layers or leukaemic serum. *J. Cell. Physiol.*, **69**, 93.

METCALF, D., NOSSAL, G. J. V., WARNER, N. L. & 4 others (1975) Growth of B lymphocyte colonies *in vitro*. *J. Exp. Med.*, **142**, 1534.

METCALF, D. (1977) *Hemopoietic Colonies*. Berlin: Springer-Verlag.

PLUZNIK, D. H. & SACHS, L. (1965) The cloning of normal "mast" cells in tissue culture. *J. Cell. Physiol.*, **66**, 319.

SHERIDAN, J. W. & METCALF, D. (1973) A low molecular weight factor in lung conditioned medium stimulating granulocyte and monocyte colony formation *in vitro*. *J. Ccll. Physiol.*, **81**, 11.

SHOHAM, D., BEN DAVID, E. & ROZENSZAJN, L. A. (1974) Cytochemical and morphologic identification of macrophages and eosinophils in tissue cultures of normal human bone marrow. *Blood*, **44**, 221.

SMITH, F., GRENAN, M. & OWENS, J. (1960) Effect of a transplanted plasma cell tumor on antibody formation. *J. Natl Cancer Inst.*, **25**, 803.

WILLIAMS, N., JACKSON, H., SHERIDAN, A. P. C., MURPHY, M. J., ELSTE, A. & MOORE, M. A. S. (1978) Regulation of megakaryopoiesis in long term murine bone marrow cultures. *Blood*, **51**, 245.